# Proposed Changes in the Physicians’ Mutual Benefit Association of Texas

**Published:** 1888-03

**Authors:** E. P. Vollum

**Affiliations:** Medical Director U. S. A., San Antonio, Texas


### PROPOSED CHANGES IN THE PHYSICIANS' MUTUAL BENEFIT ASSOCIATION OF TEXAS.

LETTER FROM DR. E. P. VOLLUM, MEDICAL DIRECTOR U. S. A., SAN ANTONIO, TEXAS.

SAN ANTONIO, TEXAS, January 14, 1888.

*Dr. F. E. Daniel:*

DEAR SIR:—I thank you for your valuable letter of the 28th ult., concerning the "Physicians Mutual Benefit Association of Texas." My object in writing to you on this subject was to learn about the system adopted by the organization, as I have studied life insurance on the association plan for some years past, and I look into every society of the kind that comes to my attention, for I believe that such fraternal organizations, business co-operations and the like are going to play a decidedly beneficent part toward ameliorating the suffering and discontent in life. After studying the system adopted by a great number of the associative benefit societies, I took from them that was useful for me, and I organized the "Army Mutual Aid Association," from which a simular society in the navy has copied, and both of them have been in successful operation for ten years and are steadily growing in numerical strength; they have dispensed a great amount of benefit and are as firmly established as the Government itself, for they have that as their foundation. I mention these instances to show that the associative plan, with a solid backing, is enduring. They are as much so as the support they rest upon. In fact, the associative plan, like your society for instance, must, to be enduring and prosperous, rest upon some permanent organization, from which it is an offshoot or dependency, with one umbilical connection, so to speak, through which it draws its strength from the parent body. The benefit societies that are offshoots of the Masons, Odd Fellows, Western Union, N. Y. Metropolitan Police, etc., etc., are permanent institutions because their parent institutions are so. And in order to inspire the confidence of the members, the officers of the parent institution usually act in similar capacity in the benefit society that is attached to it; for instance, the president of the W. U. Telegraph Co. acts as president of the WesternUnion Mutual Aid Association, so also as to the Masons, Odd Fellows, New York Police, etc.; prominent members of these orders hold office in the benefit societies connected with them. Now, applying these reflections to your society, it would appear that if it were the Mutual Benefit Association of the Texas State Medical Association, instead of resting for support only upon its charter, it would possess a more wide and solid foundation, and better chances for success.

Very truly Yours,

E. P. VOLLUM.

